# Proceedings of the Syracuse Hahnemannian Club

**Published:** 1888-12

**Authors:** Frederick Hooker

**Affiliations:** Secretary


### PROCEEDINGS OF THE SYRACUSE HAHNEMANNIAN CLUB.

At a regular meeting of Syracuse Hahnemannian Club, held July 13th, were present Drs. Brewster, Hawley, A. B. Kinne, Schumacher, True, Sheldon, Robinson, and Hooker.

On motion a committee of three was appointed by the Chair, consisting of Drs. Hawley, Robinson, and Sheldon, to draw up resolutions on the death of Mrs. Stephen Seward. (The committee reported the resolutions, which were published in the September number HOMŒOPATHIC PHYSICIAN. The resolutions were accepted and the committee discharged.)

Paragraphs 49 to 52, inclusive, of the *Organon* were read and discussed, after which Dr. Brewster gave a talk on Carbo-veg. and cited several cases.

CASE I.—Typhoid fever which had been running for two weeks, at the end of which time the patient was taken with violent hemorrhage from the bowels; laid down in the bed like a dead person; cold, almost pulseless; rational; legs as far as the knees white and cold as marble and powerless. Carbo-veg. cured.

CASE II.—A lady, æt. forty, had painless aphonia; had not talked for four weeks. Gave Carbo-veg. and "she has talked ever since!"

CASE III.—Metastasis of mumps to testicles. Carbo-veg. cured.

CASE IV.—A woman had mumps, suppressed. Sickness at stomach; heaviness of stomach; hoarseness, almost aphonia. Carbo-veg. cured.

Dr. Candee—The flatulency of Carbo-veg. is in the stomach.

Dr. Hawley—Carbo-veg. has always disappointed me in collapse.

Dr. True—A man, æt. eighty, had pneumonia and had been in a comatose condition for twelve hours. Carbo-veg. high, cured.

Dr. Sheldon—Gangrene of foot; tendons exposed. A poultice of charcoal and brewer's yeast, with Carbo-veg. internally, cured. An old lady, æt. seventy-three, had an obstinate eczema with burning pain. Ars., Rhus, and Sulph. did no good. She now complained of a burning pain in the stomach, without thirst. Carbo-veg. cured.

Dr. Hawley—Carbo-veg. has acidity of stomach, with great burning.

Dr. Schumacher spoke of a case of typhoid where Carbo-veg.$^{12x}$ relieved the tympanitis.

Dr. Brewster—Carbo-veg. has epistaxis from every effort, as straining at stool; abdomen distended, almost to bursting; incarcerated flatulence.

Dr. Hawley—I think the distention greater under Carbo-veg. than under Lycopodium.

Adjourned.

---

At the regular meeting, held July 27th, were present Drs. Seward, Hawley, True, A. B. Kinne, Schumacher, Sheldon, Robinson, and Hooker.

Paragraph 61 of the *Organon* was read.

Dr. Hawley—That paragraph disposes of the doctrine of palliation.

Dr. Robinson—Does not a high potency act longer than a low potency?

Dr. Seward—I think we get the best cures from the high.

Dr. Hawley—It seems to me that modern physicians of our school do not observe this paragraph, or they would not give cathartics in constipation. If all who believe in Homœopathy appreciated this paragraph they would be better homœopathists.

Dr. Candee—Do old-school physicians cure disease?

Dr. Hawley—It is by Homœopathy, if they do. Patients often get well in spite of treatment and the physician gets the credit of curing them.

Dr. Candee—Is the law of Homœopathy universal?

Dr. Hawley—If drugs are curative they are curative according to law. The allopaths claim to be guided by "experience." We claim to have a law, and thirty years of practice make me *know* that we have *the* law. The average allopathic death-rate in all acute diseases is twelve out of every hundred; homœopathic death-rate, four out of every hundred.

Dr. Candee then gave the following indications for Ignatia: Functional disturbances of the nervous system; the key-notes to its selection being found in the mental sphere. There is depression; variable mood; disposition to silent brooding; fullness of head, relieved by stooping. Cough, with sensation as if the larynx would close. Empty, weak feeling at stomach; flat taste. Paradoxical symptoms in fever.

Dr. Hawley—Dr. Candee's remark about the allopaths regarding Ignatia and Nux as identical shows how wise they are in therapeutics. They never learn anything because they never give their drugs to any but the sick. Have used Ignatia in nervous dyspepsia, with a "gone feeling" at stomach relieved for the time by eating, but soon returning. The Ignatia patient is not so easily angered as the Nux patient. Have verified the cough symptoms many times.

Dr. Seward—Cured a case of prolapsus ani where there was prolapse with every stool, with Ignatia.

Dr. Kinne—Case of intermittent fever. During chill, thirst; wanted to be uncovered; during heat no thirst, wanted to be covered. Ignatia cured.

Dr. True—One marked indication for Ignatia is great mental depression after losing some one who was very dear.

Adjourned to August 3d.

---

At the meeting, held August 3d, were present Drs. Seward, Hawley, True, Leggett, Robinson, Schumacher, and Hooker.

Paragraphs 62 to 66, inclusive, of the *Organon* were read.

Dr. True—An allopath once said to me, "You always give drugs until you get drug effect, do you not?"

Dr. Hooker—When I give Opium$^{30}$ in constipation, the relaxation does not take place until the reaction sets in.

Dr. Hawley—People should learn that every drug affects the vitality and *is not* harmless, as is supposed.

Dr. True—When you get a proving do you let it alone?

Dr. Leggett—An aggr. often means a bad prescription. When you get an aggr. from a remedy, without any amel., antidote it.

Dr. Hawley then gave the following indications for Colocynthis: Tearing, cutting pains; relief of colic from pressure; left-sided pains in general; left-sided prosopalgia; left-sided sciatica; stools watery, greenish-yellow, or yellow.

Colocynthis is a long acting remedy.

Dr. Leggett—Relief from heat and hard pressure. Pains indicate inflammation of nerve-sheaths.

Dr. Seward—Feeling as if squeezed between two stones; vehement person, who won't answer questions. Cures colic from cold.

Dr. Hooker—Relief of colic after stool.

Dr. True—A woman had profuse menstruation suddenly suppressed, pulse 120, temperature 105°, fearful pains, Coloc.$^{200}$, was given, and she soon began to flow freely.

Dr. Seward—I think we seldom *know* that our remedies have cured.

Adjourned to August 10th.

---

At the regular meeting, held August 10th, were present Drs. Seward, Hawley, Schumacher, Leggett, True, and Hooker.

Paragraphs 67 and 68 of the *Organon* were read.

Dr. Hawley—It is not the remnant of the drug disease that is left. The remedy removes the disease, but the vitality having been depressed, requires time to regain its former vigor.

Dr. Leggett—There is sometimes a drug force left.

Dr. Hooker then gave the following indications for Phosphorus: Hoarseness, worse mornings; cough, hard, dry, shaking whole body, loose, without expectoration, with soreness of chest and pain in head. Aggr. laughing, talking, eating, cold air, at night. Sense of weight on the chest. Rumbling in abdomen; chronic, painless diarrhœa, worse mornings. Constipation, stools dry and slender, like a dog's. Urine profuse, watery, pale; or scanty, hot, high-colored, in jaundice.

CASE—M. D., æt. seventy-five, blue eyes, spare habit, stoop-shouldered. Was attacked seven years ago with diarrhœa, which became chronic; worse mornings, driving out of bed. Stools profuse, running away from her, painless. Sulph.$^{30}$ controlled the disease for about three weeks, when it recurred and was afterward partially controlled by Sulph.$^{200}$, Thuja$^{30}$, Cinch.$^{30}$, Phos.$^{200}$, in repeated doses, during the next three years. Last January, I sent her one powder of Phos.$^{200}$ and Sac. lac. The effect was almost magical, and she is now almost well and can eat things which would have harmed her before she took the Phos., without feeling the worse for it. She has had two or three doses since January.

CASE—Little girl, æt. seven years, brown eyes, light-brown hair. Troubled with worms, grinding teeth at night, and chewing the tongue, restlessness and moaning. Took a severe cold one week before I saw her, and now had a dry, spasmodic cough, which seemed hard enough to shake her to pieces. Cina$^{30}$, four doses. Worm symptoms disappeared and the cough became loose and rattling, but *without* expectoration. Left tonsil swollen and extending half-way across the fauces, and there was a large ulcerated patch thereon, larger than the end of a lead pencil. No pain, irritation, or soreness was felt in the throat. Phos.$^{30}$, one dose was given, about six P. M., and in one hour a high fever came up, which lasted until morning, *but the cough was much better*. At one P. M., next day, she received another dose of Phos.$^{30}$, which, as before, was followed by fever, which lasted until the next morning. She received no more medicine, but the next day the fever began at one P. M., and lasted until eight P. M., when it left, and she recovered rapidly.

Dr. Seward—A married woman came to me to be treated for leucorrhœa. Phos.$^{6}$, cured, and she soon became pregnant.

Dr. Hawley—Have cured consumption with the 80 M (F.).

Dr. Leggett—Characteristic of Phos. are weakness, trembling, and irritability.

Adjourned to August 17th.

FREDERICK HOOKER, *Secretary.*

